# OTUD5 Regulates p53 Stability by Deubiquitinating p53

**DOI:** 10.1371/journal.pone.0077682

**Published:** 2013-10-15

**Authors:** Judong Luo, Zhonghua Lu, Xujing Lu, Ling Chen, Jianping Cao, Shuyu Zhang, Yang Ling, Xifa Zhou

**Affiliations:** 1 Department of Radiotherapy, Changzhou Tumor Hospital, Soochow University, Changzhou, China; 2 School of Radiation Medicine and Protection and Jiangsu Provincial Key Laboratory of Radiation Medicine and Protection, Soochow University, Suzhou, China; German Cancer Research Center, Germany

## Abstract

**Background:**

The p53 tumour suppressor protein is a transcription factor that prevents oncogenic progression by activating the expression of apoptosis and cell-cycle arrest genes in stressed cells. The stability of p53 is tightly regulated by ubiquitin-dependent degradation, driven mainly by its negative regulators ubiquitin ligase MDM2.

**Principal Findings:**

In this study, we have identified OTUD5 as a DUB that interacts with and deubiquitinates p53. OTUD5 forms a direct complex with p53 and controls level of ubiquitination. The function of OTUD5 is required to allow the rapid activation of p53-dependent transcription and a p53-dependent apoptosis in response to DNA damage stress.

**Conclusions:**

As a novel deubiquitinating enzyme for p53, OTUD5 is required for the stabilization and the activation of a p53 response.

## Introduction

p53, often regarded as the “guardian of the genome”, exerts tumor suppressive capacities by centrally coordinating a regulatory circuit that monitors and responds to a variety of stress signals, including DNA damage, abnormal oncogenic events, telomere erosion and hypoxia [1,2]. p53 is a sequence-specific transcription factor and responds to these stress events via regulating cell cycle progression, apoptosis, DNA repair, senescence, cellular metabolism or autophagy[3]. Recently studies suggest that unconventional activities of p53, such as metabolic regulation and antioxidant function, are critical for suppression of early-onset spontaneous tumorigenesis[4]. 

In order to coordinate a wide variety of cellular processes, p53 demands a refined and complicated regulatory network consisting of many positive and negative regulators. At homeostasis, the steady-state level of p53 is kept low and p53 function is repressed mainly by the negative regulators Human Double Minute 2 (HDM2, mouse ortholog is mdm2) and Human Double Minute X (HDMX, mouse ortholog is mdmX) [3]. Under stress conditions, however, p53 is stabilized and released from repression, and further activated in a promoter-specific fashion.

Protein ubiquitination is a reversible process and several families of enzymes have been described which possess deubiquitinating activity, including the ovarian tumour proteases (OTU), ubiquitin-specific proteases (USPs), ubiquitin C-terminal hydrolases (UCHs), Machado-Joseph disease proteins (MJD) and the Jab1/MPN/Mov34 metalloenzymes (JAMM) [5]. The deubiquitinating enzymes (or DUBs) have been shown to play a role in the cleavage of ubiquitin from translational precursors and in the maintenance of free ubiquitin levels within the cell. However, DUBs can also remove both monoubiquitin and polyubiquitin chains from proteins, or can trim the distal ubiquitin from polyubiquitin chains. Consequently, these activities can potentially antagonize the functions of ubiquitination within the cell[6].

A number of DUBs have been shown to influence p53 stability and activity. The herpes virus-associated USP (HAUSP or USP7) can bind, deubiquitinate and stabilize p53[7]. However, HAUSP also deubiquitinates MDM2 and reduction of HAUSP levels, either by RNA interference or by gene deletion, produces a complex phenotype [7–10]. Deletion of the HAUSP gene or extensive knockdown by RNAi results in an almost complete loss of MDM2 and consequently significant stabilization of p53 and cell death. In contrast, a more modest reduction in HAUSP causes a decrease in both MDM2 and p53 stability, suggesting that, under these conditions, sufficient MDM2 remains to degrade p53. USP10 is a cytoplasmic DUB that relocalizes to the nucleus in response to DNA damage, where it both stabilizes p53 and prevents nuclear export of p53, so contributing to p53-mediated apoptosis[11]. USP29 has been shown to be transcriptionally induced following oxidative stress, when it contributes to the full induction of a p53 response[12]. Other DUBs involved in the p53 pathway include the MDM2-specific DUB USP2a USP42 [13,14] and USP5, which degrades K48-linked polyubiquitin chains and so indirectly regulates levels of p53[14]. 

OTUD5 belongs to a subfamily of 14 DUBs characterized by an ovarian tumor (OTU) domain. OTUD5 has been shown to suppress the type I interferon-dependent innate immune response by cleaving the polyubiquitin chain from an essential type I interferon adaptor protein. Cleavage results in disassociation of the adaptor protein from a downstream signaling complex and disruption of the type I interferon signaling cascade[15].

We screened a human bone marrow cDNA library with human p53(73-393aa ) as a bait and identified the OTUD5 as a novel deubiquitinating enzyme for p53. OTUD5 is required for the stabilization and the activation of a p53 response.

## Results

### P53 interacts with OTUD5

**Figure 1:**
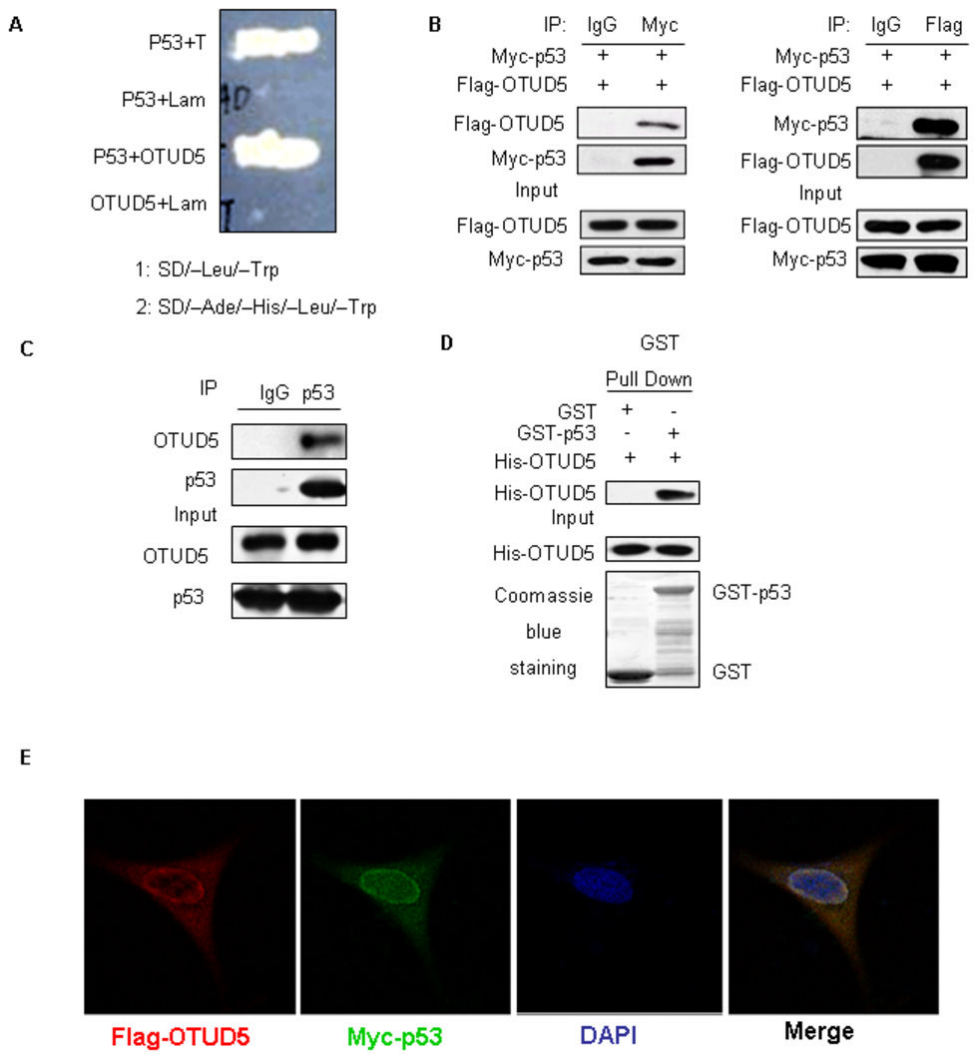
OTUD5 interacts with p53. (A) OTUD5 interacts with p53 in yeast two-hybrid assay. AH109 yeast cells were cotransformed with indicated plasmids, then plated on SD/–Ade/–His/–Leu/–Trp selection mediums. (B) The HEK293 cells were cotransfected with Flag- OTUD5 and Myc-p53, and cell lysates were immunoprecipitated with indicated antibodies and immunoblotted with anti-Myc or anti-Flag antibody. (C) Immunoprecipitation of p53 from U2OS cells, followed by western blotting of the precipitated proteins with anti- OTUD5, anti-p53 and anti-MDM2 antibodies. (D)Pull-down assay of His-tagged OTUD5 by GST fusion p53 protein bound to GSH-agarose beads, and subsequent detection by western with anti-His antibody. (E) Immunofluorescent detection of Flag-OTUD5 and Myc-p53. Nuclei are counterstained by DAPI.

To better understand the modulation of p53 activity, we sought to identify novel p53 partners using a yeast two-hybrid screen. We screened a human bone marrow cDNA library with human p53(73-393aa ) as a bait. From 2×10^6^ yeast transformants screened, about 50 putative positive clones were isolated. Among them, one clone encoding OTUD5, as confirmed by sequencing, was found to be a novel p53 interacting protein (Figure 1A), consistent with proteomic studies by the Harper Laboratory[16] that suggested an interaction of p53 with OTUD5. Most other preys we obtained such as MIZ1[17], was already reported to interact with p53 previously. Next, the interaction between p53 and OTUD5 in mammalian cells was verified by co-immunoprecipitation (Co-IP) assay. As shown in Figure 1B, Flag-OTUD5 can be observed in the Myc-p53 but not control immunoprecipitates. In a reciprocal co-IP experiment, Myc-p53 could only be pulled down together with Flag-OTUD5 but not Flag mock. These results demonstrate that p53 interacts with OTUD5 specifically in mammalian cells. Furthermore, we sought to determine whether endogenous p53 interacts with endogenous OTUD5. Indeed, OTUD5 was detected in the anti-p53 but not normal IgG immunoprecipitates from U2OS cell lysate (Figure 1C). Besides, the direct interaction between OTUD5 and p53 was performed by a GST pull-down assay. As shown in Figure 1D, GST-p53 but not GST interacted with the full-length OTUD5. We carried out localization study for both p53 and OTUD5 protein. Both proteins localized in the nucleus and cytoplasm and they co-localized very well (Figure 1E). Therefore, OTUD5 protein interacts with p53.

### OTUD5 regulates p53 turnover

**Figure 2:**
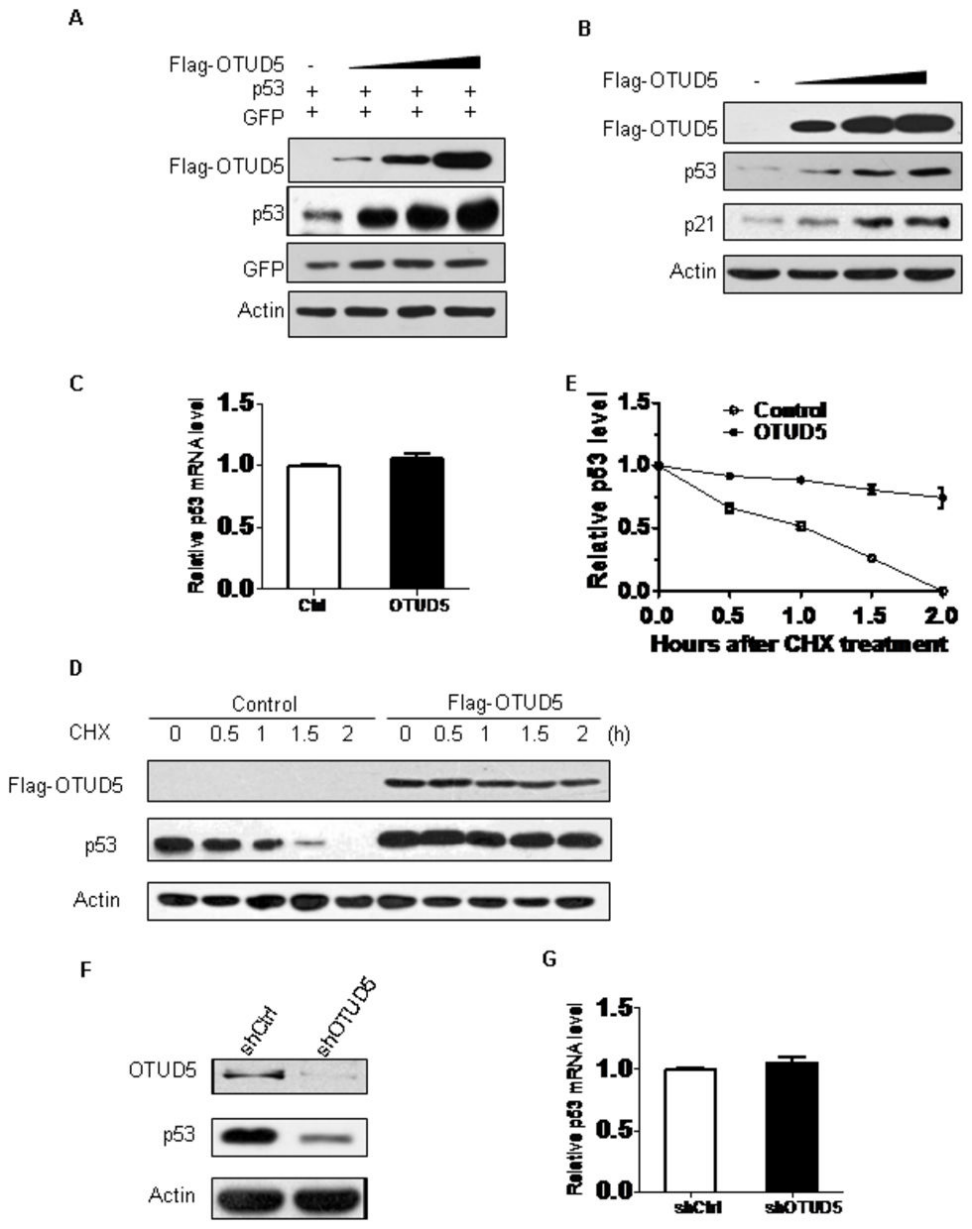
OTUD5 regulates p53 turnover. (A) The H1299 cells were cotransfected with 1 μg p53 along with increasing amounts of Flag-OTUD5. Cell lysates were prepared 24 h posttransfection and analyzed for the indicated proteins. Myc-GFP expression was used as an internal control for transfection efficiency. Actin expression shows equal loading of samples. (B) The U2OS cells were transfected with increasing amounts of Flag-OTUD5. Cell lysates were prepared 24 h posttransfection and analyzed for the indicated proteins. (C) Quantitative real time RT-PCR analysis of p53 mRNA level. RNA was extracted from U2OS-control and U2OS-OTUD5 cells. p53 mRNA was measured by quantitative RT-PCR, and levels relative to GAPDH mRNA are shown. Results are the means ± standard deviations of three independent experiments. (D) Half-life assay of endogenous p53 protein. U2OS cells were transfected with vector or Flag-OTUD5 then treated with 30 μg/ml cycloheximide for the indicated durations and protein levels were detected by western blotting using anti-p53, anti-Flag and anti-actin antibodies. (E) Quantification of the experiment. The values shown are obtained from three independent experiments and are normalized to the actin control. For each experimental condition, the signal at the start of the experiment was set to one. (F) Endogenous p53 levels in control and shOTUD5 U2OS cells were analyzed by western blotting with the anti-p53 antibody. Actin was used as loading control. (G) Quantitative real time RT-PCR analysis of p53 mRNA level. RNA was extracted from control and shOTUD5 U2OS cells. p53 mRNA was measured by quantitative RT-PCR, and levels relative to GAPDH mRNA are shown. Results are the means ± standard deviations of three independent experiments.

During our studies, we consistently observed that the level of p53 is higher in cells transfected with OTUD5 than in cells transfected with empty vector. Therefore, we explored the possibility that OTUD5 could regulate the abundance of p53. To this end, 1μg of p53 and increasing amounts of Flag-OTUD5 were cotransfected into H1299 cells. As shown in Figure 2A, overexpression of OTUD5 resulted in a dose-dependent increase in p53 levels. In p53-positive U2OS cells we also found the accumulation of p53 with a concomitant increase in p21 level (Figure 2B). Quantitative real time RT-PCR analysis revealed that p53 mRNA levels remain unchanged after OTUD5 expression (Figure 2C), suggesting that the increased p53 levels observed in OTUD5 expressing cells might be due to enhanced p53 protein stability. Therefore, we performed protein half-life assay to examine the stability of p53 in U2OS cells expressing OTUD5. Cells were treated with cycloheximide (CHX) to block protein synthesis and cell lysates were prepared at different times after CHX treatment. As shown in Figure 2D and E, p53 stability was increased in cells transfected with OTUD5. Using shRNA-mediated depletion of OTUD5 in U2OS cells caused marked decrease of p53 protein levels with no effect on p53 mRNA levels (Figure 2F and G). These results suggest that OTUD5 regulates p53 stability.

### OTUD5 deubiquitininates p53

**Figure 3:**
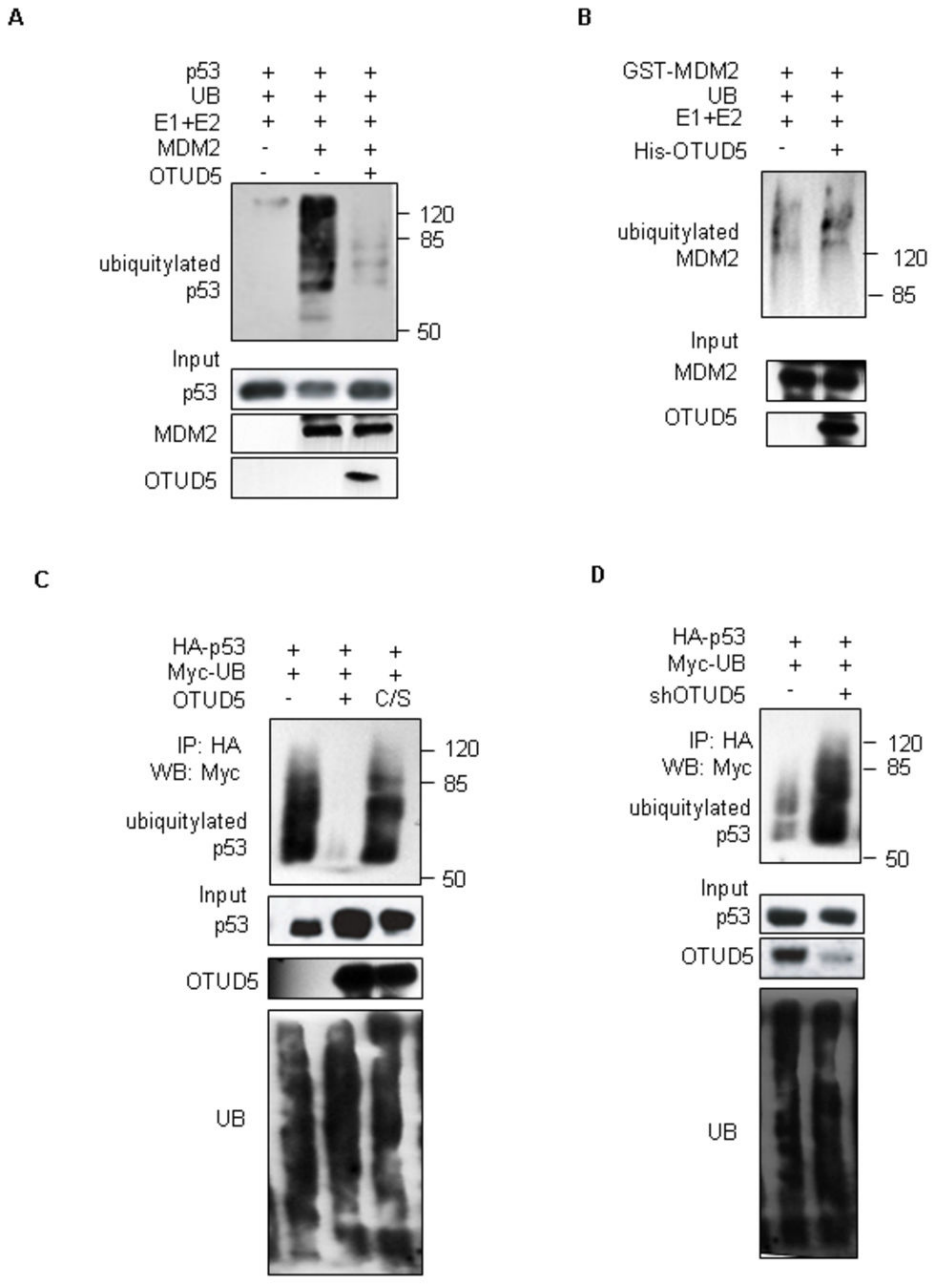
OTUD5 deubiquitininates p53. (A) In vitro translated p53 was pre-incubated for 90 min with a bacterially expressed MDM2 and subsequently immunoprecipitated with an anti-p53 antibody. Immunoprecipitated p53 was then incubated with in vitro translated OTUD5, followed by western blotting with anti-UB to detect the ubiquitination of p53 and blotted with anti-p53, anti-OTUD5 and anti-MDM2 as loading control. (B) OTUD5 does not affect the auto-ubiquitination of MDM2 in vitro. Immobilized GST–Mdm2 protein was incubated with His-OTUD5, E1, E2 and Ub as indicated. (C,D) The H1299 cells were transfected with the indicated combinations of expression vectors. Cells, 24 h after transfection, were treated with MG132 (5 μM) for 3 h and then lysed. Cell lysates were immunoprecipitated with anti-HA antibody (against HA-p53) and immunoblotted with anti-Myc antibody (against Myc-ubiquitin).

OTUD5 is a deubiquitinase that deubiquitinates substrates. We speculated that p53 is a OTUD5 substrate. We examined the effect of adding OTUD5 to p53 that had been ubiquitinated by MDM2 in vitro (Figure 3A). This assay showed that while OTUD5 efficiently removed ubiquitin from p53. To verify that the deubiquitination of p53 by OTUD5 is specific, MDM2 was also tested in the in vitro deubiquitination assay. As shown in Figure 3B, MDM2 cannot be deubiquitinated by OTUD5. We also investigated whether OTUD5 is necessary for p53 ubiquitination in vivo. To address this, we carried out an in vivo ubiquitination assay. H1299 cells were transfected with constant amounts of p53, ub together with OTUD5. Thereafter, cells were harvested, p53-ubiquitin conjugates were immunoprecipitated with HA antibody and detected by a Western blotting assay with Myc antibody. As shown in Figure 3C, a similar reduction of MDM2-dependent ubiquitination of p53 was seen following co-transfection of OTUD5 into H1299 cells. We also found that catalytically inactive OTUD5(C224S) did not affect ubiquitination of p53.Moreover, we transfected H1299 cells stably expressing OTUD5 shRNA and the control cells with HA-p53 and Myc-ub, followed by MG132 treatment. P53 ubiquitination was significantly increased in OTUD5 knockdown cells compared with the control cell (Figure 3D). Overall, OTUD5 deubiquitinates p53, leading to the stabilization of p53.

### OTUD5 can help in the stabilization and activation of p53 in response to DNA damage

**Figure 4:**
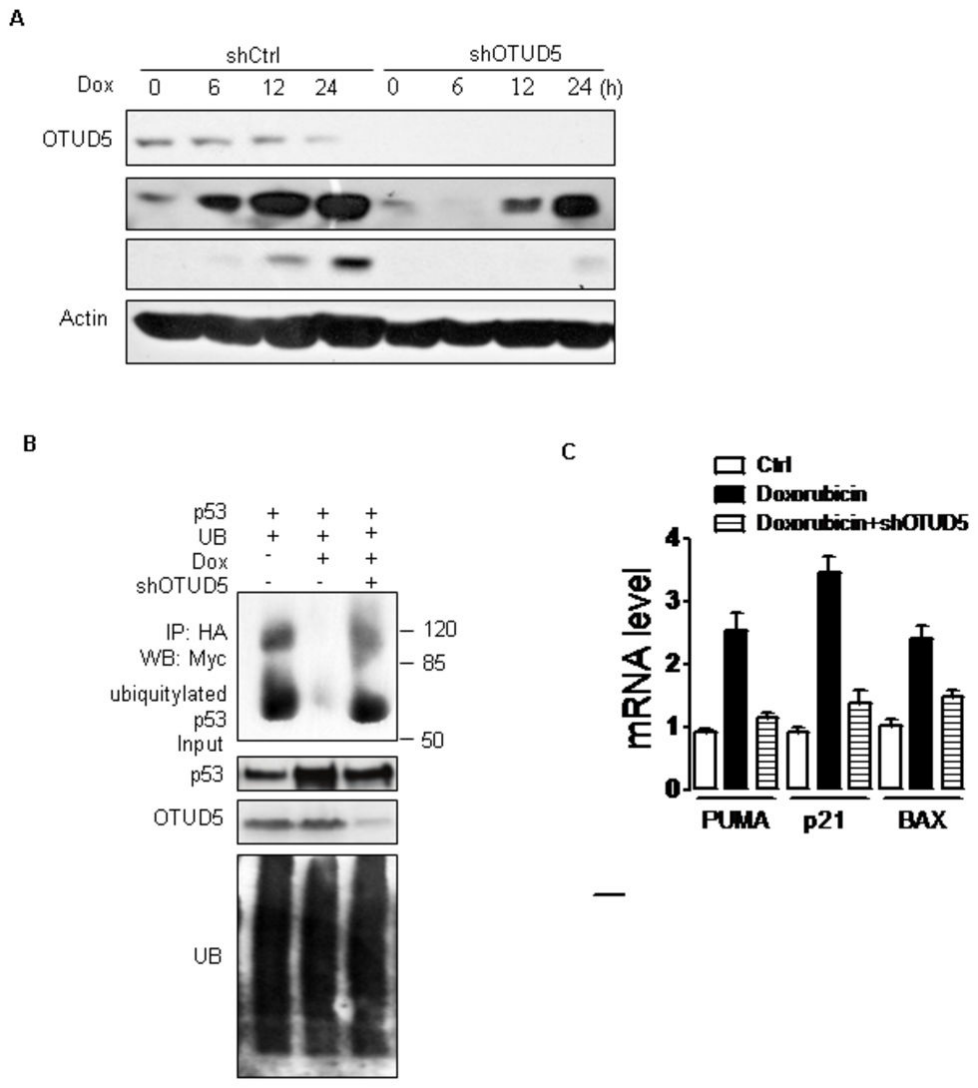
OTUD5 can help in the stabilization and activation of p53 in response to DNA damage. (A) U2OS cells were infected with lentiviruses expressing either control or shRNA targeting OTUD5. After 48 h, cells were treated with 2.5μM Dox for indicated time. Cells were harvested and lysates were prepared for western blotting. The panels show immunoblots probed with the indicated antibodies. (B) U2OS-control and U2OS-shRNA cells were transfected with indicated combinations of expression vectors. Cells, 24 h after transfection, were treated with 2.5μM Dox for 3 h and MG132 (5 μM) for 3 h, then lysed. Cell lysates were immunoprecipitated with anti-HA antibody (against HA-p53) and immunoblotted with anti-Myc antibody (against Myc-ubiquitin). (C) Expression of Puma p21 Bax mRNA by quantitative RT-PCR. U2OS-control and U2OS-OTUD5-shRNA cells were treated with 2.5μM Dox for 12 h, and mRNA were extracted and subjected to quantitative RT-PCR.

p53 is stabilized in response to stress, and so we considered a role for OTUD5 during the activation of p53 by DNA damage treatments. Doxorubicin was reported to induce DNA damage[18]. U2OS cells transfected with scramble shRNA or OTUD5 shRNA were treated with Doxorubicin. p53 was stabilized after treatment of cells with Doxorubicin. In these experimental conditions, Doxorubicin-induced p53 expression was largely abrogated in cells silenced for OTUD5 (Figure 4A). In concert with this result, mRNA expression of p53-responsive genes, such as Puma Bax and p21, were also attenuated in the absence of OTUD5 (Figure 4C). Moreover, we examined the consequences of OTUD5 down regulation on p53 ubiquitination during the the DNA damage stress response. As shown in Figure 4 after Doxorubicin treatment depletion of OTUD5 clearly prevented the efficient deubiquitination of p53 that is associated with protein accumulation (Figure 4B). Our data show that OTUD5 can help in the stabilization and activation of p53 in response to DNA damage.

### OTUD5 is required for p53-induced apoptosis in response to DNA damage

**Figure 5:**
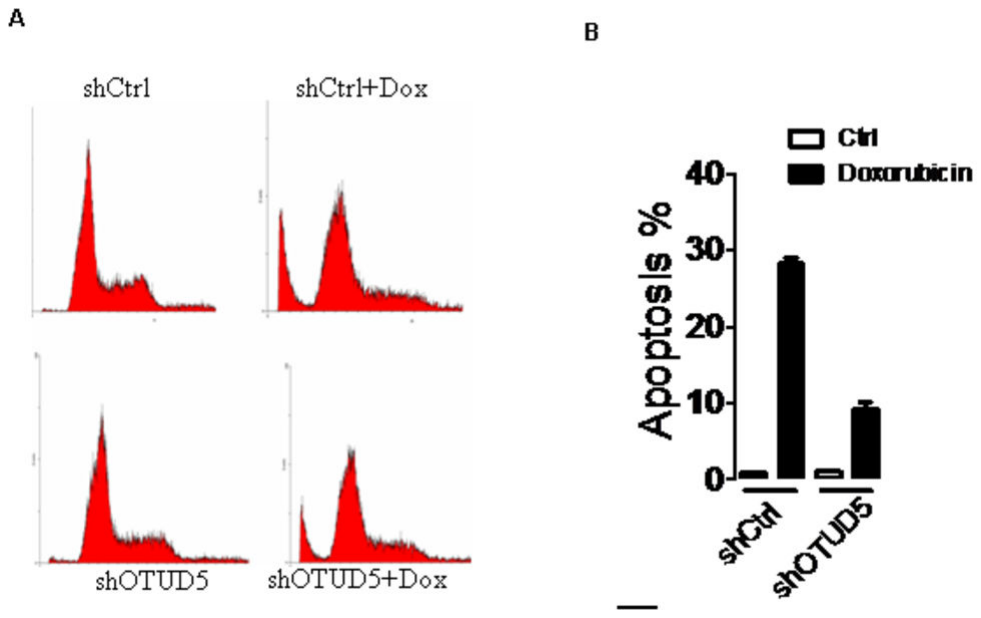
(A,B) FACS analysis. U2OS-control and U2OS-OTUD5-shRNA cells were treated with or without 2.5μM Dox as indicated. 24 h later, cells were harvested, stained with propidium iodide (PI), and the cell cycle distribution was determined by flow cytometry. The percentage of cells in various phases of the cell cycle is shown.

To look more closely at the effect of OTUD5 depletion on cell growth and survival, we examined apoptosis of wild-type p53 expressing U2OS cells in response to Doxorubicin treatment. After treatment of control cells with Doxorubicin resulted in a clear accumulation of apoptosis cells (Figure 5A and B) reflecting the activation of p53. Knockdown of OTUD5 clearly impeded the apoptosis, consistent with a weaker activation of p53 (Figure 4A). Overall, OTUD5 regulates p53-dependent DNA damage response.

## Discussion

One of the principal mechanisms by which p53 activity is regulated is through the control of protein stability. This has been shown to be mediated by proteasome- dependent degradation following the ubiquitination of p53[19]. Many ubiquitin ligases that target p53 have been described[20–26], with MDM2 playing a predominant role both in controlling basal levels of p53 in normal unstressed cells and in re-establishing low levels of p53 following the resolution of a stress response. Deubiquitinating enzymes that cleave ubiquitin chains can also control the extent of ubiquitination of p53, and therefore its sensitivity to degradation. Recent studies have identified a number of DUBS that may help to stabilize p53.

In this study, we provide new evidences that OTUD5 can also function to deubiquitinate and stabilize p53. We show that (1) OTUD5 is a novel p53 interacting protein; (2) OTUD5 deubiquitinates p53, leading to the stabilization of p53; (3) OTUD5 can help in the stabilization and activation of p53 in response to DNA damage; (4) OTUD5 is required for p53-induced apoptosis in response to DNA damage. These results reveal that OTUD5 is required for the stabilization and the activation of a p53 response.

It has been reported that OTUD5 could suppress the type I interferon-dependent innate immune response by cleaving the polyubiquitin chain from an essential type I interferon adaptor protein TRAF3. Cleavage results in disassociation of the adaptor protein from a downstream signaling complex and disruption of the type I interferon signaling cascade[15]. In this study, we found that OTUD5 directly suppresses MDM2-mediated p53 ubiquitination in cells and in vitro, leading to stabilization and activation of p53. To verify that the deubiquitination of p53 by OTUD5 is specific, MDM2 was also tested in the in vitro deubiquitination assay and found thatMDM2 cannot be deubiquitinated by OTUD5 (Figure 3B).

Other recently described DUBs that can target p53 have been shown to be active under selective conditions. USP10 is normally cytoplasmic, and is driven to the nucleus where it can affect p53 localization and stability in response to DNA damage[11]. USP29 is induced by JTV1 and FBP-driven transcription in response to oxidative stress, and was also shown to be important for the full activation of PUMA and apoptosis[12]. It has been reported that USP42 interacts with and deubiquitinates p53. USP42 forms a direct complex with p53 and controls level of ubiquitination during the early phase of the response to a range of stress signals including Dox [14].. Our results that OTUD5 is important for p53 stabilization and activation after DNA damage provide another regulation route.

Deubiquitinating enzymes that cleave ubiquitin chains can also control the extent of ubiquitination of subtracts, and therefore its sensitivity to degradation. Our data have shown that catalytically inactive OTUD5(C224S) did not affect ubiquitination of p53. So we can draw a conclusion that OTUD5 could cleave ubiquitin chains from p53 and control the extent of ubiquitination of subtracts, and therefore its sensitivity to degradation. Our data show that Doxorubicin-induced p53 expression was largely abrogated in cells silenced for OTUD5 (Figure 4A). However, the underlying mechanism is not clear. It is possible that the the OTUD5-p53 and MDM2-p53 associations are modulated in response to DNA damage response. Another possibility is that stress induced modification of OTUD5 could alter activity towards p53.

In conclusion, our work revealed that OTUD5 plays an important role in p53 in response to DNA damage stress. 

## Materials and Methods

### Yeast two-hybrid screen

PGBKT7-p53(73-393aa ) was used to screen a human bone marrow MATCHmaker cDNA library cloned into pACT2 (Clontech) according to the manufacturer’s instructions. Briefly, the yeast strain AH109 was sequentially transformed with the pGBKT7- p53(73-393aa ) vector and the library. An estimated 10^6^ transformants were screened. Yeasts containing interacting proteins were identified by growth on selective media lacking leucine, tryptophan, and histidine and confirmed by β-galactosidase activity. Plasmids harboring interacting cDNAs were recovered from yeast by reintroduction into *E.coli*, and the identities of putative interacting proteins were determined by sequencing and BLAST search of the NCBI database.

### Expression vectors

Full-length p53 and OTUD5 MDM2 were amplified by PCR and subcloned into pCMV-Myc/HA, pGEX-4T, pGBKT7 vector (Clontech) pCMV-Tag2 vector (Stratagene). All constructs derived from PCR products were verified by DNA sequencing.

### Cell culture, transient transfection and treatments

The H1299, U2OS, HEK293 and 293T cell lines were obtained from the American Type Culture Collection. Human H1299, U2OS, HEK293 and 293T cells were cultured in DMEM (Gibco) supplemented with 10% fetal bovine serum (Hyclone), 100 μg/ml penicillin and streptomycin. Transient transfections were carried out using standard calcium phosphate method. Doxorubicin, MG132 and cycloheximide were purchased from Sigma, and added to subconfluent cells at the indicated doses. 

### RNA interference

For RNA interference experiments we used a lentivirus-based vector pLL3.7. Oligonucleotides targeting OTUD5 (gcggctgctgctccgggcc) were cloned into pLL3.7 vector. Recombinant lentiviral plasmids were co-transfected into 293T cells with the packaging plasmids VSV-G, RSV-REV and pMDL, and after 48 h the viral supernatants were used to infect target cells in the presence of 6 μg/ml polybrene (Sigma).

### Western blotting and Immunoprecipitation

Total cell extracts were prepared in cell lysis buffer (20 mM Tris-HCl [pH7.5], 150 mM NaCl, 10 mM NaF, 20 mM β-glycerophosphate, 1 mM sodium orthovanadate, 1 mM PMSF, 10 μg/ml leupeptin, 2 μg/ml aprotinin, 1% Triton X-100, and 1 mM EDTA) for immunoprecipitations. Immunocomplexes were resolved by SDS-PAGE, and western blotting was performed with the following antibodies: anti-Flag M2 and anti-actin monoclonal antibodies (Sigma); HA (Y-11), anti-Myc (9E10), OTUD5 and p53(DO1) monoclonal antibodies (Santa Cruz Biotechnology); HRP-conjugated goat anti-mouse, anti-rabbit (Thermo). 

### GST Pull-down Assays

*E. coli* BL21 cells harboring expression vectors for GST-p53, His-OTUD5, or GST alone were grown to an A600 nm of 0.6–0.8 and induced with IPTG for 6h. Purified His- OTUD5 from *E. coli* BL21 was incubated with GST or GST-p53 bound to glutathione–Sepharose beads. Beads were collected by centrifugation and washed five times with phosphate-buffered saline. Proteins retained on the beads were then blotted with the His antibody.

### Immunofluorescence staining

U2OS cells grown on coverslips in sixwell plates were transfected with Flag-OTUD5 and Myc-p53. After 36h, cells were washed once with PBS and fixed with 4% formaldehyde in PBS for 10 min at room temperature. Cells were then rinsed with PBS, before being treated with PBS, 0.2% Triton X-100, 2 mg/ml BSA and 1 mM NaN_3_ on ice for 10 min. The coverslips were then incubated with PBS, 0.02% Triton X-100, 3% BSA and 1 mM NaN_3_ for 30 min. Cells were then incubated with 2 μg/ml Flag (OctA Santa Cruz D-8) and Myc (Santa Cruz 9E10) antibody in blocking solution for 4h. Next, cells were washed four times with PBS, 0.02% Triton X-100, 1.5% BSA and 1 mM NaN_3_; incubated with 20 μg/ml of goat anti-rabbit Alexa Fluor 594 (Invitrogen) and goat anti-mouth-FITC (Invitrogen) in blocking solution for 1h. Cells were then washed four times with PBS, 0.02% TritonX‑100, 1.5% BSA and 1 mM NaN_3_ before being incubated with DAPI (1 μg/ml) in washing solution for 2 min. Specimens were mounted in 90% glycerol and sealed with nail polish.

### In vivo ubiquitination assays

The cells were cotransfected with expression vectors of p53, Myc-ubiquitin, OTUD5. 36 h after transfection, cells were treated with MG132 (5 μM) for 3 h then lysed with RIPA buffer (1% SDS, 0.5% sodium deoxycolate, 0.5% Nonidet P-40, 10 mM NaF, 20 mM β-glycerophosphate, 1 mM sodium orthovanadate, 1 mM PMSF, 10 μg/ml leupeptin and 2 μg/ml aprotinin). After boiling the cell lysis for 5min, dilute the SDS concentrate to 0.1%, HA-p53 was immunoprecipitated with anti-HA antibody and then western blot analyzed with anti-Myc antibody (against Myc-ubiquitin). 

### In-vitro deubiquitination assays

The in-vitro ubiquitination of p53 by MDM2 was carried out as described previously [27]. Subsequently, in-vitro translated and precipitated GST-OTUD5 was added, followed by incubation at 37°C for 1 h. The reaction was stopped by adding 3*SDS loading buffer and was analysed by 8% SDS–PAGE, followed by immunoblotting with UB antibody (Santa Cruz).

### Quantitative real-time RT-PCR

Total RNA was isolated from cells using RNA Simple Total RNA kit (TIANGEN), and first-strand cDNA was synthesized from lμg of total RNA using the First-Strand cDNA Synthesis kit (TOYOBO) following the manufacturer’s instructions. Prepared cDNA samples were amplified and analyzed by quantitative real-time PCR with Power SYBR Green PCR Master Mix (Applied Biosystems) using ABI 7500 Real Time PCR System (Applied Biosystems). Primer sequences were as follows: human p21 forward, 5-GGCCCGGAACATCTCAGG-3; p21 reverse, 5-AAATCTGTCAGGCTGGTCTGC-3;Puma forward, 5-TCACCCTGGAGGGTCATGTA-3; Puma reverse, 5-GCGGGTGTAGGCACCTAGT-3; Bax forward, 5-TGGAGATGAACTGGACAGCA-3; Bax reverse, 5-GAAGTTGCCATCAGCAAACA-3; p53 forward, 5-ACTGCATGGACGATCTGTTG-3; and p53 reverse, 5-GTGACAGGGTCCTGTGCTG-3. Each sample was assessed in triplicate. Relative mRNA levels were normalized to the housekeeping GAPDH mRNA and calculated using the comparative threshold cycle method (2-ΔΔ^Ct^).

### Flow cytometry

Cells were washed with PBS and fixed at 4°C with 70% ethanol. Cells were then washed with PBS and incubated in PBS containing 100 μg/ml RNaseA at 37°C for 30 minutes, stained with propidium iodide at 4°C overnight. DNA content was analyzed with a FACS Diva Flow Cytometer (Beckman).

## References

[1] Horn HF, Vousden KH (2007) Coping with stress: multiple ways to activate p53. Oncogene 26: 1306-1316. doi:10.1038/sj.onc.1210263. PubMed: 17322916. 17322916

[2] Vousden KH, Lane DP (2007) p53 in health and disease. Nat Rev Mol Cell Biol 8: 275-283. doi:10.1038/nrm2147. PubMed: 17380161. 17380161

[3] Kruse JP, Gu W (2009) Modes of p53 regulation. Cell 137: 609-622. doi:10.1016/j.cell.2009.04.050. PubMed: 19450511. 19450511 PMC3737742

[4] Li T, Kon N, Jiang L, Tan M, Ludwig T et al. (2012) Tumor suppression in the absence of p53-mediated cell-cycle arrest, apoptosis, and senescence. Cell 149: 1269-1283. doi:10.1016/j.cell.2012.04.026. PubMed: 22682249. 22682249 PMC3688046

[5] Nijman SM, Luna-Vargas MP, Velds A, Brummelkamp TR, Dirac AM et al. (2005) A genomic and functional inventory of deubiquitinating enzymes. Cell 123: 773-786. doi:10.1016/j.cell.2005.11.007. PubMed: 16325574. 16325574

[6] Komander D, Clague MJ, Urbé S (2009) Breaking the chains: structure and function of the deubiquitinases. Nat Rev Mol Cell Biol 10: 550-563. doi:10.1038/nrm2731. PubMed: 19626045. 19626045

[7] Li M, Brooks CL, Kon N, Gu W (2004) A dynamic role of HAUSP in the p53-Mdm2 pathway. Mol Cell 13: 879-886. doi:10.1016/S1097-2765(04)00157-1. PubMed: 15053880. 15053880

[8] Cummins JM, Vogelstein B (2004) HAUSP is required for p53 destabilization. Cell Cycle 3: 689-692. PubMed: 15118411. 15118411

[9] Li M, Chen D, Shiloh A, Luo J, Nikolaev AY et al. (2002) Deubiquitination of p53 by HAUSP is an important pathway for p53 stabilization. Nature 416: 648-653. doi:10.1038/nature737. PubMed: 11923872. 11923872

[10] Meulmeester E, Maurice MM, Boutell C, Teunisse AF, Ovaa H et al. (2005) Loss of HAUSP-mediated deubiquitination contributes to DNA damage-induced destabilization of Hdmx and Hdm2. Mol Cell 18: 565-576. doi:10.1016/j.molcel.2005.04.024. PubMed: 15916963. 15916963

[11] Yuan J, Luo K, Zhang L, Cheville JC, Lou Z (2010) USP10 regulates p53 localization and stability by deubiquitinating p53. Cell 140: 384-396. doi:10.1016/j.cell.2009.12.032. PubMed: 20096447. 20096447 PMC2820153

[12] Liu J, Chung HJ, Vogt M, Jin Y, Malide D et al. (2011) JTV1 co-activates FBP to induce USP29 transcription and stabilize p53 in response to oxidative stress. EMBO J 30: 846-858. doi:10.1038/emboj.2011.11. PubMed: 21285945. 21285945 PMC3049210

[13] Stevenson LF, Sparks A, Allende-Vega N, Xirodimas DP, Lane DP et al. (2007) The deubiquitinating enzyme USP2a regulates the p53 pathway by targeting Mdm2. EMBO J 26: 976-986. doi:10.1038/sj.emboj.7601567. PubMed: 17290220. 17290220 PMC1852834

[14] Hock AK, Vigneron AM, Carter S, Ludwig RL, Vousden KH (2011) Regulation of p53 stability and function by the deubiquitinating enzyme USP42. EMBO J 30: 4921-4930. doi:10.1038/emboj.2011.419. PubMed: 22085928. 22085928 PMC3243628

[15] Kayagaki N, Phung Q, Chan S, Chaudhari R, Quan C et al. (2007) DUBA: a deubiquitinase that regulates type I interferon production. Science 318: 1628-1632. doi:10.1126/science.1145918. PubMed: 17991829. 17991829

[16] Sowa ME, Bennett EJ, Gygi SP, Harper JW (2009) Defining the human deubiquitinating enzyme interaction landscape. Cell 138: 389-403. doi:10.1016/j.cell.2009.04.042. PubMed: 19615732. 19615732 PMC2716422

[17] Miao L, Song Z, Jin L, Zhu YM, Wen LP et al. (2010) ARF antagonizes the ability of Miz-1 to inhibit p53-mediated transactivation. Oncogene 29: 711-722. doi:10.1038/onc.2009.372. PubMed: 19901969. 19901969

[18] Cox RF (1984) Managing skin damage induced by doxorubicin hydrochloride and daunorubicin hydrochloride. Am J Hosp Pharm. 41(11): 2410-2414. PubMed: 6150636. 6150636

[19] Dai C, Gu W (2010) p53 post-translational modification: deregulated in tumorigenesis. Trends Mol Med 16: 528-536. doi:10.1016/j.molmed.2010.09.002. PubMed: 20932800. 20932800 PMC2978905

[20] Grossman SR, Deato ME, Brignone C, Chan HM, Kung AL et al. (2003) Polyubiquitination of p53 by a ubiquitin ligase activity of p300. Science 300: 342-344. doi:10.1126/science.1080386. PubMed: 12690203. 12690203

[21] Haupt Y, Maya R, Kazaz A, Oren M (1997) Mdm2 promotes the rapid degradation of p53. Nature 387: 296-299. doi:10.1038/387296a0. PubMed: 9153395. 9153395

[22] Kubbutat MH, Jones SN, Vousden KH (1997) Regulation of p53 stability by Mdm2. Nature 387: 299-303. doi:10.1038/387299a0. PubMed: 9153396. 9153396

[23] Münger K, Howley PM (2002) Human papillomavirus immortalization and transformation functions. Virus Res 89: 213-228. doi:10.1016/S0168-1702(02)00190-9. PubMed: 12445661. 12445661

[24] Shi D, Pop MS, Kulikov R, Love IM, Kung AL et al. (2009) CBP and p300 are cytoplasmic E4 polyubiquitin ligases for p53. Proc Natl Acad Sci U S A 106: 16275-16280. doi:10.1073/pnas.0904305106. PubMed: 19805293. 19805293 PMC2752525

[25] Chen D, Kon N, Li M, Zhang W, Qin J et al. (2005) ARF-BP1/Mule is a critical mediator of the ARF tumor suppressor. Cell 121: 1071-1083. doi:10.1016/j.cell.2005.03.037. PubMed: 15989956. 15989956

[26] Brooks CL, Gu W (2006) p53 ubiquitination: Mdm2 and beyond. Mol Cell 21: 307-315. doi:10.1016/j.molcel.2006.01.020. PubMed: 16455486. 16455486 PMC3737769

[27] Uldrijan S, Pannekoek WJ, Vousden KH (2007) An essential function of the extreme C-terminus of MDM2 can be provided by MDMX. EMBO J 26: 102-112. doi:10.1038/sj.emboj.7601469. PubMed: 17159902. 17159902 PMC1782374

